# Prevalence and influencing factors of functional constipation in Chinese children and adolescents: a systematic review and meta-analysis

**DOI:** 10.3389/fpubh.2026.1776863

**Published:** 2026-03-12

**Authors:** Qin Liu, Yue’e Dai, Zitong Zhou, Shixiu Wang, Qian Wang, Xiaozhao Gu, Xuemei Luo

**Affiliations:** Sichuan Provincial Center for Mental Health, Sichuan Provincial People's Hospital, University of Electronic Science and Technology of China, Chengdu, China

**Keywords:** adolescents, children, functional constipation, influencing factors, meta-analysis, prevalence

## Abstract

**Objective:**

In recent years, the incidence of functional constipation in children and adolescents has been increasing annually, seriously affecting their physical and mental development and quality of life, and placing a significant burden on society and families. This study aims to conduct a systematic review and meta-analysis to explore the prevalence and influencing factors of functional constipation among children and adolescents in China, providing evidence-based evidence for developing scientific intervention strategies for patients with functional constipation.

**Methods:**

We systematically searched relevant studies in databases including CNKI, Wanfang Data Knowledge Service Platform, SinoMed, VIP, PubMed, EmBase, Web of Science, and Cochrane Library from their inception until December 28, 2025. Two researchers independently screened and cross-checked the studies. The quality of cross-sectional studies was assessed using the evaluation tool from the Agency for Healthcare Research and Quality (AHRQ), and meta-analysis was conducted using Stata 17.0 software.

**Results:**

We finally included 27 studies, involving a total of 112,801 cases. The results showed that the prevalence of functional constipation among children and adolescents in China was 7.8% (95% CI (6.0, 9.9%)). Subgroup analysis demonstrated that children and adolescents aged 5–12 years, females, those residing in North China, those diagnosed according to the Rome IV criteria, and those studied from 2021 onwards had a higher prevalence of functional constipation. Risk factors for functional constipation included female, family history of constipation, inadequate water intake, low intake of vegetables and fruits, low physical activity, being scolded for poor bowel habits in early childhood, non-breastfeeding, picky eating, food allergy, obesity, and high household income. Protective factors included adequate sleep and the development of good bowel habits.

**Conclusion:**

Current evidence indicates a relatively high prevalence of functional constipation among children and adolescents in China and is impacted by diverse factors. Therefore, future initiatives should prioritize training for primary healthcare professionals, conducting health education campaigns, raising parents’ awareness of the risks associated with functional constipation in children and adolescents, reducing the prevalence of the condition, and improving the quality of life for affected children and their families.

## 1 Introduction

Functional constipation is a gastrointestinal motility disorder arising from dysregulation of the gut–brain interaction. It is one of the most common disorders during childhood and adolescence, defined as a non-organic functional impairment characterized by delayed defecation, passage of hard stools, and defecation accompanied by straining or pain under certain circumstances, in the absence of any underlying organic disease (e.g., anatomical, endocrine, neurological, or other systemic disorders) (1, 2). According to relevant literature, the prevalence of functional constipation varies by region, ranging from 0.5 to 32.2% among children and adolescents globally, with a combined prevalence of 9.5%. In China, the reported prevalence of Functional constipation in this population ranges from 3.1 to 13.7% (3, 4). Functional constipation is not only closely associated with adverse outcomes in adolescents such as poor appetite, cognitive sluggishness, memory impairment, and enuresis (5–8), but long-term functional constipation may also induce secondary conditions like anal fissures, fecal impaction, and rectal bleeding. Additionally, it can trigger psychological issues including separation anxiety, generalized anxiety, social phobia, depression, and oppositional defiant disorder, all of which negatively impact the physical and mental health of affected children (9, 10). Research indicates that approximately one-third of pediatric constipation patients continue to experience symptoms into adulthood, severely impacting their development, family relationships, and quality of life, imposing a heavy disease burden and psychological stress on both patients and their families (11). Therefore, conducting epidemiological investigations and analyses of influencing factors for functional constipation in children and adolescents is particularly necessary. Currently, no systematic review exists on functional constipation in Chinese children and adolescents, and existing studies show inconsistent results due to varying diagnostic criteria and regional differences. Based on this, we plan to employ systematic review and meta-analysis methods to systematically examine the prevalence and influencing factors of functional constipation in Chinese children and adolescents, aiming to provide clinical reference for the prevention and treatment of functional constipation in this population.

## 2 Methods

This systematic review adheres to the Preferred Reporting Items for Systematic Reviews and Meta-Analyses (PRISMA) statement (12) and is registered in PROSPERO with the registration number: CRD42024550114.

### 2.1 Search strategy

We systematically searched databases including CNKI, Wanfang, VIP, SinoMed, PubMed, Web of Science, Embase, and Cochrane Library for studies on the prevalence and influencing factors of functional constipation. The search period spanned from each database’s inception to December 28, 2025. The search employed a combination strategy of Medical Subject Headings (MeSH) and free-text terms. Keywords included: constipation, functional constipation, habitual constipation, defecation disorder, colonic motility delay, defecation difficulty, chronic constipation, functional gastrointestinal disorder, children, adolescents, Hong Kong, Taiwan, Macau, Chinese, etc. (see Supplementary File 1, which details PubMed search strategies).

### 2.2 Inclusion and exclusion criteria

The inclusion criteria were as follows: (a) study type: cross-sectional studies, cohort studies, or case–control studies; (b) study subjects: children and adolescents aged 0–18 years residing in China; (c) Clear diagnostic criteria for functional constipation were applied in this study, referencing internationally recognized standards, including the Rome III and Rome IV criteria, as well as other established diagnostic criteria; (d) outcome measures: prevalence of functional constipation and its influencing factors.

The exclusion criteria were as follows: (a) case reports, conference abstracts, and review articles; (b) full-text inaccessible or duplicated publications; (c) literature not published in Chinese or English; (d) publications with unobtainable data or missing data; (d) Secondary constipation caused by endocrine disorders, neurological disorders, metabolic disorders, or organic lesions of the colon and rectum (such as tumors or strictures); (e) Patients meeting diagnostic criteria for other functional gastrointestinal disorders, particularly irritable bowel syndrome, functional diarrhea, and functional bloating/abdominal distension.

### 2.3 Study selection and data extraction

After importing all retrieved articles into EndNote X9, two evaluators independently screened studies based on inclusion and exclusion criteria, followed by cross-checking. Any discrepancies were resolved through discussion with a third reviewer. Data extracted from the final included studies comprised: authors and publication year, region, age, sample size, sample source, prevalence, diagnostic criteria, and influencing factors. These data were compiled into an Excel spreadsheet.

### 2.4 Quality assessment

We used the assessment tool developed by the Agency for Healthcare Research and Quality (AHRQ) (13) to evaluate the methodological quality of the included cross-sectional studies. The quality assessment of the included studies was performed independently by two reviewers. The AHRQ tool comprises 11 items, with studies scoring 8–11, 4–7, and 0–3 points classified as high-quality, moderate-quality, and low-quality, respectively.

### 2.5 Statistical analysis

We employed Stata17.0 software to conduct statistical analyses of the prevalence and influencing factors of functional constipation. Effect sizes were expressed as odds ratios (*OR*) with 95% confidence intervals (*CI*). Heterogeneity was assessed using *Q* test and *I*^2^ statistics. A fixed-effects model was used when *I*^2^ < 50%, and a random-effects model when *I*^2^ ≥ 50%. Sources of heterogeneity were further explored through sensitivity and subgroup analyses. Factors that could not be quantitatively pooled were presented using descriptive analysis. Publication bias was assessed through visual inspection of funnel plots, supplemented by Begg’s test and Egger’s test (when ≥10 studies were included).

## 3 Results

### 3.1 Search results

**Figure 1:**
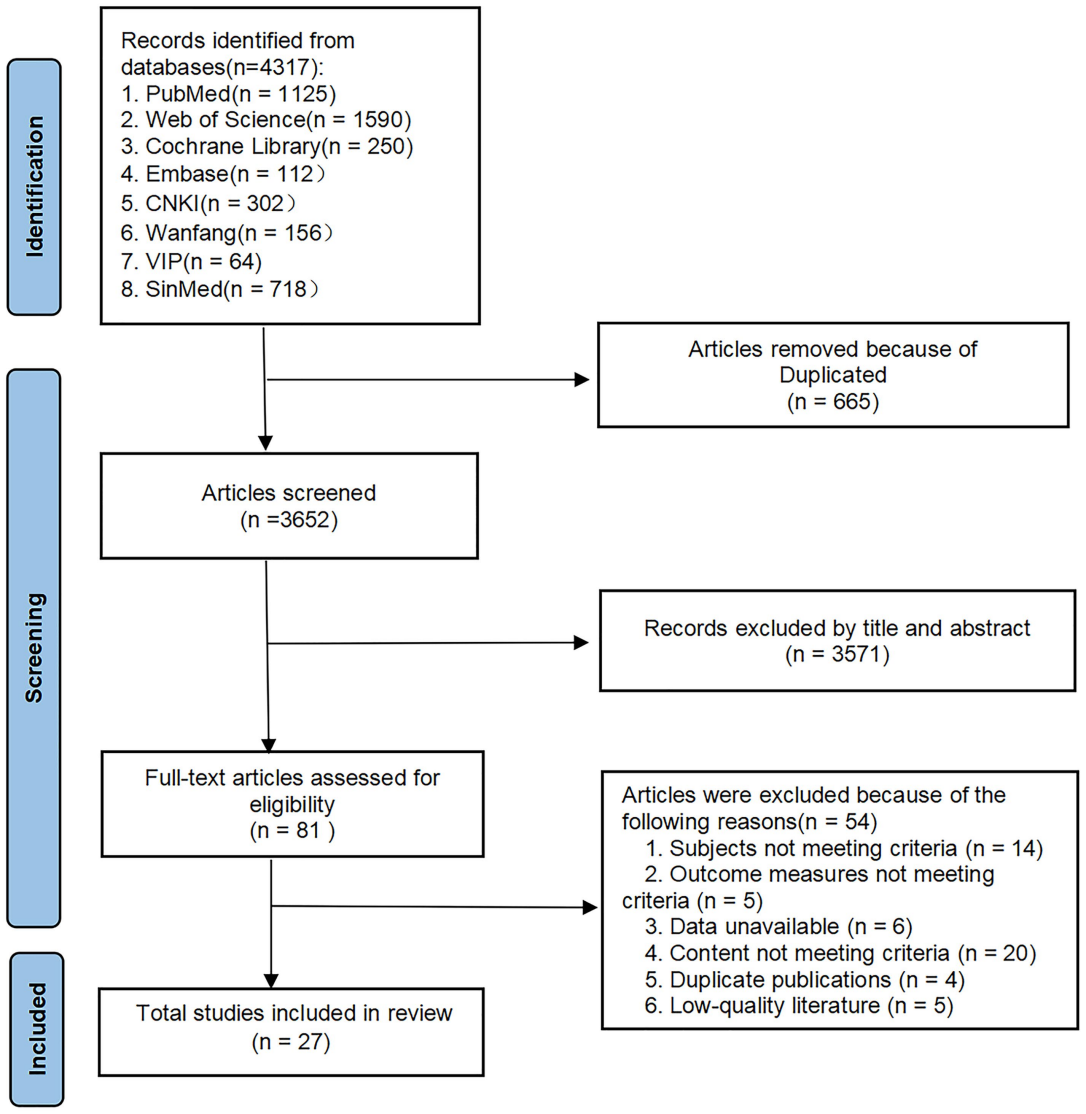
Flow diagram of study selection.

A total of 4,317 relevant studies were retrieved. After stepwise screening, we ultimately included 27 studies (3, 11, 14–38). The literature screening flowchart is shown in Figure 1.

### 3.2 Basic characteristics and quality evaluation results of included literature

Among the 27 studies included in this review, a total of 112,801 patients were involved, including 9,461 patients with functional constipation. The quality of the literature was assessed using the Agency for Healthcare Research and Quality (AHRQ) evaluation criteria. The assessment revealed that 16 studies were of high quality (3, 11, 14, 16, 24–26, 29–34, 36–38), while 11 studies were of moderate quality (15, 18–24, 27, 28, 35). Detailed results are presented in Table 1 (For comprehensive results of the literature evaluation, please refer to Supplementary File 2).

**Table 1:** General information and quality evaluation results of the included literature.

Study	Region	Population source	Age (years)	Total sample size (*n*)	Constipation cases	Diagnostic criteria	Influencing factors	Quality score
Zhang et al., 2010 (14)	Five Northern Cities	School	4 ~ 14	19,286	913	B	–	9
Zhou et al., 2010 (15)	Hunan	School	10 ~ 18	2,075	130	B	③	7
Chan et al., 2010 (16)	Hong Kong	School	8 ~ 10	383	28	C	–	9
Zhou et al., 2011 (17)	Shanghai	School	Secondary School Students	3,671	915	B	⑬	7
Xiong et al., 2011 (18)	Guangdong	School	6 ~ 13	1,786	98	B	–	7
Lv et al., 2012 (19)	Guangdong	School	4 ~ 16	5,731	400	B	②③④	6
Yan et al., 2015 (20)	Jiangsu	School	5 ~ 12	5,860	809	B	②④⑤⑧⑨⑪⑫⑬	5
Zhang et al., 2015 (21)	Shandong	Community	Primary & Secondary School Students	1,650	98	B	–	4
Wu et al., 2016 (22)	Gansu	School	Secondary School Students	764	41	B	–	7
Zhao et al., 2017 (23)	Henan	School	Secondary School Students	528	27	B	–	4
Ji et al., 2018 (24)	Seven Provinces	Hospital	0 ~ 4	20,932	1,755	A	–	8
Zhou et al., 2018 (25)	Guangdong, Henan	School	3 ~ 6	5,146	201	B	–	8
Chen et al., 2019 (26)	Sichuan	Hospital	0 ~ 4	9,826	609	B	③⑧⑨⑩⑪⑫	8
Yang et al., 2020 (27)	Yunnan	Hospital	4 ~ 16	300	20	B	②③④⑤⑦	5
Wang et al., 2020 (28)	Fujian	Hospital	0 ~ 4	1,006	26	A	–	6
Huang et al., 2021 (29)	Shanghai, Zhejiang	Hospital	0 ~ 4	2,604	92	A	–	8
Li et al., 2022 (30)	Shaanxi	Hospital	0 ~ 4	2,615	260	A	③	8
Zhuang et al., 2022 (31)	Shanghai	School	Secondary School Students	4,969	693	A	⑥	8
Zhang et al., 2022 (3)	Beijing	Hospital	0 ~ 4	1,264	125	A	①⑧⑩	9
Lin et al., 2022 (32)	Fujian	Hospital	0 ~ 4	2,397	90	A	③⑮	8
Huang et al., 2022 (33)	Fujian	Hospital	0 ~ 4	1,006	36	A	⑮	9
Yang et al., 2023 (34)	Shaanxi	School	0 ~ 18	9,133	364	A	①⑧	8
Xu et al., 2023 (35)	Inner Mongolia	Hospital	0 ~ 14	4,374	1,238	A	②③④⑤⑦⑭	5
Zhou et al., 2023 (36)	Anhui	Community	0 ~ 18	832	94	A	③⑥⑨	9
Wang et al., 2024 (11)	Qinghai	School	6 ~ 12	1,253	132	B	③⑨⑩⑭	8
Gong et al., 2025 (37)	Guizhou	Community	0 ~ 4	2,039	151	A	–	8
Yin et al., 2025 (38)	Ningxia	School	13 ~ 15	1,371	106	A	⑤	8

Functional Constipation Diagnostic Criteria: A is the “Rome IV Criteria for Functional Gastrointestinal Disorders in Children” (39, 40); B is the “Rome III Criteria for Functional Gastrointestinal Disorders in Children” (41, 42); C is the “Constipation Assessment Scale (CAS)” (43). Influencing Factors: ① Gender, ② Inadequate water intake, ③ Family history of constipation, ④ Low intake of vegetables and fruits, ⑤ Insufficient physical activity, ⑥ Adequate sleep, ⑦ Being scolded for poor bowel habits in early childhood, ⑧ Non-breastfed, ⑨ Picky eating or dietary preference, ⑩ Food allergy, ⑪ Obesity, ⑫ High household income, ⑬ Frequent consumption of fried foods, ⑭ Development of good bowel habits, ⑮ Probiotic supplementation after birth.

### 3.3 Meta-analysis results

#### 3.3.1 Meta-analysis of the prevalence of functional constipation in Chinese children and adolescents

**Figure 2:**
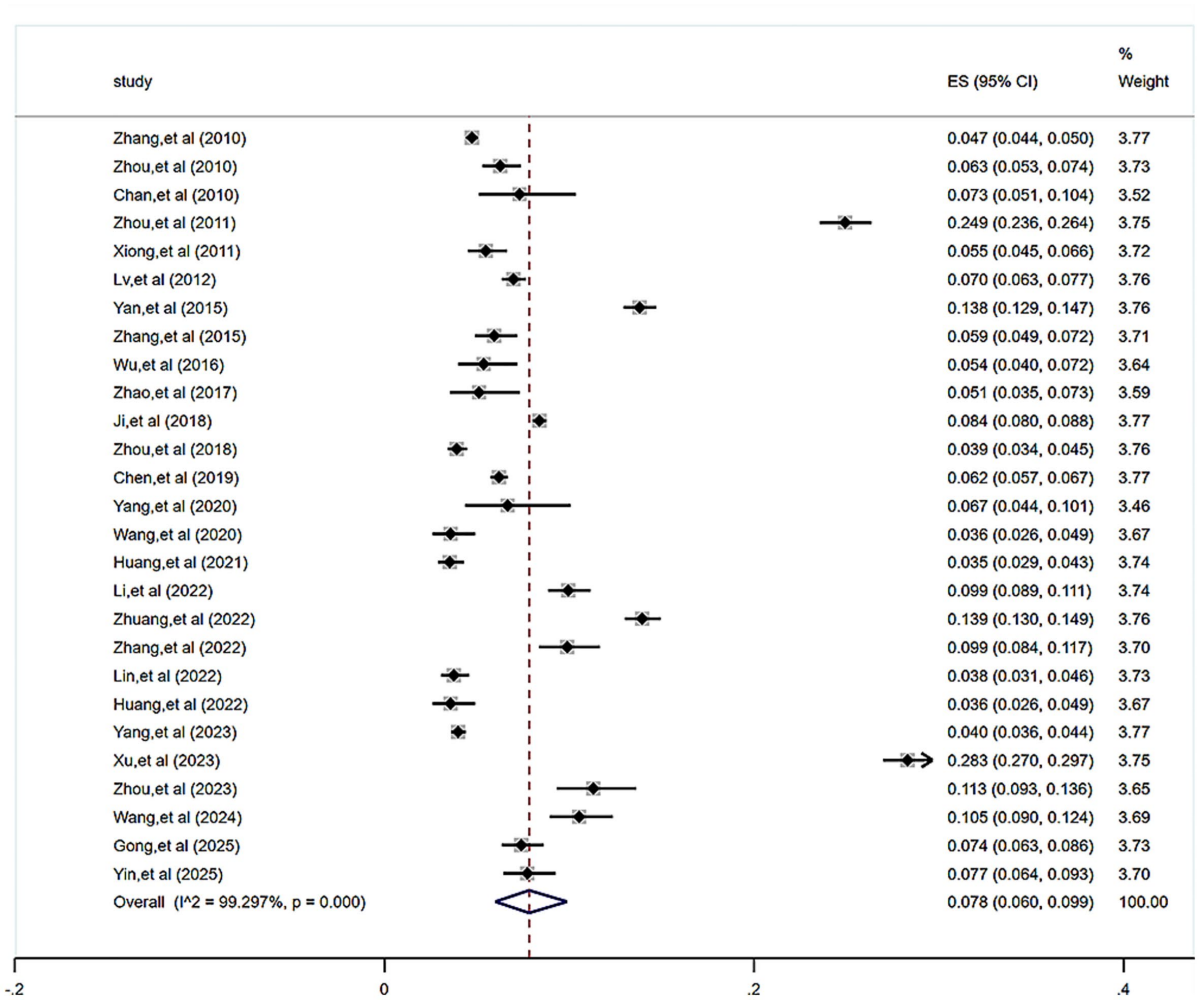
Forest plot of the prevalence of functional constipation in Chinese children and adolescents.

In a total of 27 studies, we investigated the prevalence of functional constipation among children and adolescents, involving 112,801 children and adolescents. The results of the meta-analysis indicated significant heterogeneity among the studies (*I*^2^ = 99.30%, *p* < 0.001). Therefore, a random-effects model was employed for the meta-analysis. The results showed a pooled functional constipation prevalence rate of 7.8% (95% CI (6.0, 9.9%)), as shown in Figure 2.

#### 3.3.2 Subgroup analysis of the prevalence of functional constipation in Chinese children and adolescents

Meta-analysis revealed high heterogeneity in our studies. Subgroup analyses were subsequently conducted to explore potential sources of clinical heterogeneity, based on factors including sex, age, region, diagnostic criteria, and study publication year. The results indicated that the prevalence of functional constipation was significantly higher (*p* < 0.001) in subgroups comprising children and adolescents who were aged 5–12 years, female, residing in North China, diagnosed using the Rome IV criteria, or studied from 2021 onward. The detailed results of the subgroup analyses are presented in Table 2.

**Table 2:** Subgroup analysis of prevalence of functional constipation in Chinese children and adolescents.

Subgroup	Number of included literature	Results of heterogeneity test		Effect model	The prevalence % (95%Cl)
		*I^2^*(%)	*p*		
Age					
0 ~ 4 (3, 20, 21, 22, 24–26, 28–33)	13	99.41	<0.001	Random effect mode	7.2 (4.7, 10.2)
5 ~ 12 (7, 10–18, 21, 23, 27, 30–32)	16	99.56	<0.001	Random effect mode	9.2 (6.0, 12.9)
13 ~ 18 (10, 11, 13–15, 17–19, 23, 27, 30–32, 34)	14	99.57	<0.001	Random effect mode	8.9 (5.5, 13.1)
Sex					
Male (3, 7, 10–14, 16, 17, 22, 26–28, 30, 32–34)	17	98.31	<0.001	Random effect mode	8.0 (5.8, 10.4)
Female (3, 7, 10–14, 16, 17, 22, 26–28, 30, 32–34)	17	98.48	<0.001	Random effect mode	9.7 (7.2, 12.6)
Region					
Central China (11, 19)	2	–	–	Random effect mode	6.0 (5.1, 7.0)
South China (12, 14, 15)	3	–	–	Random effect mode	6.5 (5.3, 7.7)
East China (13, 16, 17, 24, 25, 27–29, 32)	9	99.35	<0.001	Random effect mode	8.4 (4.5, 13.4)
North China (3, 31)	2	–	–	Random effect mode	23.6 (22.5, 24.7)
Northwest China (7, 18, 26, 30, 34)	5	97.72	<0.001	Random effect mode	7.3 (4.4, 10.8)
Southwest China (22, 23, 33)	3	–	–	Random effect mode	6.6 (5.7, 7.6)
Diagnostic criteria					
Rome IV (3, 20, 24–34)	13	99.41	<0.001	Random effect mode	8.1 (5.2, 11.6)
RomeIII (7, 10, 11, 13–19, 21–23)	13	99.24	<0.001	Random effect mode	7.6 (5.2, 10.5)
Constipation assessment scale (12)	1	–	–	Random effect mode	7.3 (5.1, 10.4)
Study publication year					
Before 2021 (10–24)	15	99.14	<0.001	Random effect mode	7.2 (5.2, 9.4)
2021 onward (3, 7, 25–34)	12	99.45	<0.001	Random effect mode	8.7 (5.0, 13.3)

### 3.4 Meta-analysis of influencing factors of functional constipation Chinese children and adolescents

We pooled studies with ≥2 common associated factors. Results indicate that female sex, family history of constipation, inadequate water intake, low intake of vegetables and fruits, low physical activity, being scolded for poor bowel habits in early childhood, non-breastfeeding, picky eating, food allergy, obesity, and high household income were associated factors for functional constipation in children and adolescents(*P* < 0.05). Conversely, adequate sleep and the development of good bowel habits were identified as protective factors against functional constipation in this population (*p* < 0.05). Furthermore, analysis revealed that frequent consumption of fried foods and probiotic supplementation after birth were not significant associated factors for functional constipation in children and adolescents. The results of the meta-analysis of influencing factors of functional constipation are shown in Table 3. Meanwhile, Zhou et al. (17) identified anxiety and depression as influencing factors for functional constipation in children and adolescents, and Wang et al. (11) noted that toilet training is closely associated with functional constipation in this population. Furthermore, other factors influencing functional constipation include mode of delivery (3), parental education level (20), complementary feeding (26), family discord (27), sedentary behavior (38), and types of staple foods (34), among others. Due to the limited number of included studies, only a descriptive analysis was conducted. Finally, we conducted a sensitivity analysis on the influencing factors of functional constipation. Pooled odds ratios (*OR*) with 95% confidence intervals (*CI*) were calculated using both fixed-effects and random-effects models. The results indicated that, except for non-breastfeeding and frequent consumption of fried foods, other outcomes showed no or minimal differences. This suggests that the findings of this meta-analysis are relatively stable. The sensitivity analysis of the influencing factors is presented in Supplementary File 3.

**Table 3:** Meta-analysis of influencing factors of functional constipation Chinese children and adolescents.

Influencing factors	Number of included literature	Results of heterogeneity test		Effect model	Result	
		*I*^2^(%)	*P*		*OR*(95%CI)	*P*
Female (3, 30)	2	0.0	0.641	Fixed effect model	1.40 (1.04, 1.90)	0.029
Family history of constipation (7, 11, 15, 22, 23, 26, 28, 31, 32)	9	73.1	<0.001	Random effect model	2.72 (2.03, 3.64)	<0.001
Inadequate water intake (15, 16, 23, 31)	4	83.3	<0.001	Random effect model	3.32 (2.02, 5.47)	<0.001
Low intake of vegetables and fruits (12, 16, 23, 31)	4	98.1	<0.001	Random effect model	4.52 (1.77, 11.56)	0.002
Iow physical activity (16, 23, 31, 34)	4	88.5	<0.001	Random effect model	2.75 (1.19, 6.35)	<0.001
Adequate sleep (27, 32)	2	0.0	0.461	Fixed effect model	0.58 (0.38, 0.88)	0.010
Being scolded for poor bowel habits in early childhood (23, 31)	2	0.0	0.645	Fixed effect model	3.23 (2.21, 4.71)	<0.001
Non-breastfeeding (3, 16, 22, 30)	4	92.7	<0.001	Random effect model	5.09 (1.82, 14.26)	0.002
Picky eating (7, 16, 22, 32)	4	66.6	0.029	Random effect model	2.79 (1.71, 4.56)	<0.001
Food allergy (3, 7, 22)	3	66.7	0.050	Random effect model	3.45 (1.20, 9.92)	0.022
Obesity (16, 22)	2	0.0	0.496	Fixed effect model	2.28 (1.33, 3.89)	0.003
High household income (16, 22)	2	0.0	0.581	Fixed effect model	1.50 (1.07, 2.10)	0.019
Frequent consumption of fried foods (13, 16)	2	96.5	<0.001	Random effect model	3.70 (0.75, 18.27)	0.108
Development of good bowel habits (7, 31)	2	0.0	0.757	Fixed effect model	0.54 (0.43, 0.68)	<0.001
Probiotic supplementation after birth (28, 29)	2	91.8	<0.001	Random effect model	2.20 (0.86, 5.62)	0.100

### 3.5 Sensitivity analyses and publication bias

**Figure 3:**
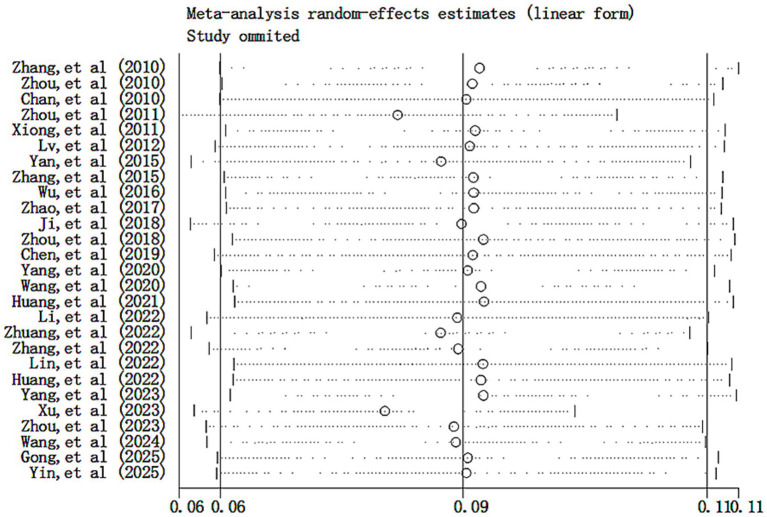
Sensitivity analysis of the prevalence of functional constipation in Chinese children and adolescents.

**Figure 4:**
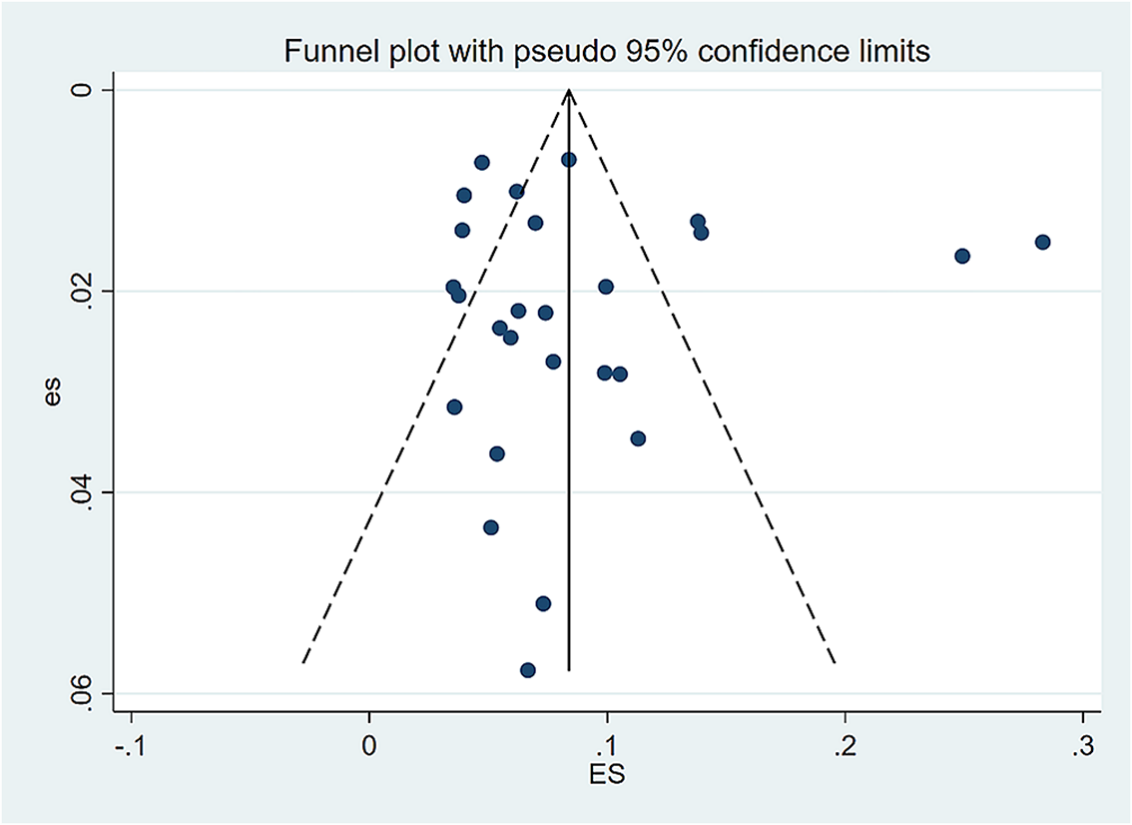
Funnel plot of the prevalence of functional constipation in Chinese children and adolescents.

A sensitivity analysis of the prevalence of functional constipation was performed using the leave-one-out method. The results showed that the pooled effect size did not change significantly after sequentially removing individual studies, indicating that the results calculated by the random-effects model were relatively stable, as shown in Figure 3. The funnel plot for studies on the prevalence of functional constipation was essentially symmetrical, with most studies concentrated at the top, as shown in Figure 4. This suggests a low likelihood of publication bias. Further assessment using Egger’s test (*p* = 0.650 > 0.05) and Begg’s test (*p* = 0.327 > 0.05) on the included studies also indicated no significant publication bias.

## 4 Discussion

### 4.1 Prevalence of functional constipation in children and adolescents

This study included 27 pieces of research. Meta-analysis results showed that the prevalence rate of functional constipation among Chinese children and adolescents was 7.8%, which is higher than the average functional constipation prevalence in Asia (6.3%) but lower than that in Western countries (Central and South America 13.3%, Europe and North America 12.4%) (4). Subgroup analysis revealed significant differences in the prevalence of functional constipation among children and adolescents based on age, sex, region, diagnostic criteria, and study publication year. Our study found: ① Age: The prevalence of functional constipation in the 5–12-year-old group (9.2%) was similar to that in the 13–18-year-old group (8.9%), both higher than that in the 0-4-year-old group (7.2%). The reasons may be that the 5–12 and 13–18 age groups correspond to school-age and adolescence, respectively, during which individuals face greater academic pressure and reduced sleep duration (44, 45). Meanwhile, changes in lifestyle, diet, interpersonal interactions, and other aspects may lead to endocrine, hormonal, or even psychological alterations contributing to functional constipation (46). Alternatively, differences in gut microbiota and function across age groups may arise with aging and dietary changes. ② Sex: The prevalence of functional constipation in females (9.7%) was higher than in males (8.0%), this gender disparity may be associated with lower levels of physical activity among females. According to data from the World Health Organization, insufficient physical activity is more prevalent in Chinese female children and adolescents than in their male counterparts, and they spend significantly more time on sedentary behaviors per day than boys (47). Of note, sedentary behavior has been demonstrated to be associated with an increased risk of constipation (48). Currently, the correlation between sex and constipation remains debated, with many studies indicating a higher functional constipation prevalence among female children than male children (49, 50). ③ Region: Subgroup analysis indicated that the prevalence of functional constipation was highest in North China (23.6%). This may be associated with inter-regional differences in economic culture, dietary habits, and climate (51, 52). ④ Diagnostic Criteria: The prevalence of functional constipation diagnosed using Rome III criteria (7.6%) was lower than that using Rome IV criteria (8.1%), indicating a considerable discrepancy between the two. This difference may be attributed to variations in the diagnostic criteria themselves, such as differences in diagnostic thresholds and symptom assessment. Research by Baaleman et al. (53) demonstrated that the prevalence diagnosed with Rome III was significantly lower, and the consistency between Rome III and Rome IV in diagnosing functional gastrointestinal disorders was limited, especially for functional constipation, irritable bowel syndrome, and functional dyspepsia. Currently, the Rome IV diagnostic criteria are most widely applied in China. To enhance the comparability of research findings and their clinical guidance value, it is recommended to uniformly adopt this standard system in subsequent functional constipation-related diagnostic and epidemiological studies. ⑤ Sudy Publication Year: The prevalence of functional constipation 2021 onward (8.7%) was higher than that before 2021 (7.2%). This may be related to the number and quality of included literature as well as the sequelae of COVID-19 (54, 55). Studies have shown that many patients experience persistent constipation symptoms after recovering from COVID-19 (56). This may be related to the mechanism by which SARS-CoV-2 causes gut microbiome dysbiosis. The virus can invade the gut during infection, leading to damage to intestinal structure and disruption of the intestinal epithelial barrier, thereby promoting intestinal inflammation and microbial dysbiosis (57). On the other hand, it may also be associated with the impact of mandatory social self-isolation on people’s lifestyles, forcing them to adopt unhealthy dietary habits, sedentary behavior, online learning, and sleep disturbances (58).

### 4.2 Influencing factors of functional constipation in children and adolescents

#### 4.2.1 Sociodemographic factors

The results of this study indicate that sex, a family history of functional constipation, and higher household income are influencing factors for functional constipation in children and adolescents. Sex is an influencing factor, with females being more prone to constipation than males, a finding consistent with Koppen et al. (4). The reasons why females are more susceptible to functional bowel disorders remain unclear, which may be associated with their tendency to restrict staple food intake, engage in excessive dieting for weight control, and have lower levels of physical activity (3). Second, there may be biological differences in intestinal motility. Compared with boys, boys’ intestinal motility appears to be more sensitive to physical activity, whereas girls’ intestines may be more responsive to psychological stress via the brain–gut axis (59). Furthermore, hormonal influences during female adolescence, where hormonal fluctuations during menstruation can inhibit colonic motility, may also contribute to constipation (60). A family history of constipation is also an influencing factor. Research by Peeters et al. (61) demonstrated that having at least one affected relative yields an odds ratio of 2.02, which increases to 3.99 with at least two affected relatives. Vriesman et al. (62) indicated a high correlation between functional constipation and familial inheritance, with children having a significantly greater risk if one parent is affected. Therefore, screening children and adolescents with a family history of functional constipation for timely diagnosis and early intervention is crucial. The study also found that higher household income is an influencing factor. This may be linked to the more refined and high-protein diets common in higher-income households (26). Research by Yan et al. (63) suggests that an overly refined diet deficient in dietary fiber results in insufficient mass and volume of fecal matter in the large intestine. This weakens colonic peristalsis, prolongs intestinal transit time, leads to excessive water absorption, and ultimately causes constipation through the formation of dry, hard stools.

#### 4.2.2 Disease-related factors

Disease-related factors associated with functional constipation identified in this study include food allergies and obesity. Food allergies in children and adolescents increase the risk of functional constipation. Studies suggest (26) that constipation in allergic children may be related to inflammatory responses in the intestinal mucosa. Intestinal inflammation can slow down intestinal motility, thereby contributing to constipation. Additionally, Scaillon et al. (64) reported that allergies can cause eosinophil infiltration in the rectal wall, leading to intestinal dysmotility and altered mucus composition, which in turn can induce functional constipation. Connor et al. (65) also noted a significantly higher incidence of functional constipation among children with food allergies. Obesity was also identified as an influencing factor. Obese children often have low dietary fiber intake, reduced physical activity, and an imbalanced diet, which increases the prevalence of functional constipation (66). Moreover, excessive accumulation of abdominal wall fat in obese children can exert pressure on the intestines, impairing normal peristalsis and defecation function, resulting in insufficient propulsive force for defecation, making it a contributing factor to constipation.

#### 4.2.3 Daily lifestyle factors

This study also identified several daily lifestyle factors associated with functional constipation in children and adolescents. Inadequate water intake and insufficient consumption of vegetables and fruits were found to contribute to constipation. Boilesen et al. (67) found a correlation between Inadequate water intake and a higher incidence of functional constipation in children. Insufficient fluid intake can lead to reduced fecal water content and harder stool consistency, thereby becoming a contributing factor to constipation. Research by Wang et al. (11) indicates an association between water/dietary fiber intake and functional constipation. Fiber interacts with water and stimulates the intestinal mucosa to increase secretion and peristalsis, promoting bowel movements. Furthermore, dietary fiber serves as a fermentation substrate for gut microbiota, promoting its proliferation. Conversely, low intake of vegetables and fruits is associated with insufficient dietary fiber, which is a contributing factor to constipation. Therefore, parents should patiently guide children to drink adequate water and consume a balanced diet rich in fruits and vegetables to prevent functional constipation. Our study also found an association between constipation and low physical activity. Song et al. (68) indicated that reduced physical activity decreases gastrointestinal motility and the secretion of digestive juices, weakening digestive and absorptive capacity and subsequently contributing to constipation. Therefore, encouraging appropriate physical activities for children, such as jogging or active play, can promote intestinal peristalsis, aid fecal expulsion, and reduce the occurrence of functional constipation. Furthermore, Tharner et al. (69) found that picky eating is associated with an increased risk of functional constipation, consistent with the results of this study. Research confirms (70) that picky eating reduces dietary diversity and fiber intake in children. Inadequate dietary fiber intake results in insufficient fecal bulk and volume in the colon, leading to inadequate colonic motility or reduced peristalsis, which in turn can induce constipation. Thus, parents need to patiently encourage children to eat a varied and balanced diet to reduce the risk of functional constipation. Adequate sleep was identified as a protective factor against functional constipation in children and adolescents. Zhou et al. (36) noted an association between sleep quality and constipation. Good sleep promotes vagus nerve excitation, which enhances intestinal peristalsis and facilitates defecation. Conversely, sleep deprivation indicates insufficient sleep duration or poor sleep quality in an individual, and chronically poor sleep quality may contribute to difficult defecation through autonomic nervous system dysfunction. Regarding bowel habits, this study found that good defecation habits can reduce the incidence of constipation and serve as a protective factor. Research by Yin et al. (71) points out that defecation is a coordinated action controlled by both the spinal primary defecation center and the cerebral cortex’s higher defecation center, involving multiple muscle groups. Therefore, conditioned reflexes can be established through training to develop good bowel habits. Feng et al. (72) found that individuals who suppress the urge to defecate or have irregular bowel movements have a significantly higher prevalence of constipation than those with good habits. Suppressing the defecation reflex can inhibit the cerebral cortex’s defecation response, reduce colonic sensitivity, lower defecation frequency, and ultimately lead to constipation.

#### 4.2.4 Early developmental factors

In this study, being scolded for poor bowel habits in early childhood and non-breastfeeding were identified as influencing factors for functional constipation. Niu et al. (73) found that children scolded for defecation issues in early childhood had a 3.788 times higher risk of functional constipation compared to those who were not. The reason may be that such children experience psychological stress and fear related to defecation. These negative emotions can regulate colonic and rectal function via efferent pathways, thereby contributing to the occurrence of constipation. Additionally, studies have shown (74) a positive correlation between non-breastfeeding and functional constipation. Lack of breastfeeding can alter the composition of the early gut microbiota, potentially with long-lasting effects. An abnormal gut microbiota plays a significant role in the pathogenesis of functional constipation and may increase the risk of developing functional constipation later in childhood, which aligns with the findings of this study.

### 4.3 Strengths and limitations

This study represents the first systematic review and meta-analysis to investigate the prevalence and influencing factors of functional constipation among children and adolescents in China. The methodological approach was rigorously designed, incorporating strict inclusion and exclusion criteria, and all included studies underwent detailed quality assessment, thereby enhancing the reliability of the findings. The review strictly adhered to PROSPERO prospective registration and the PRISMA reporting guidelines, ensuring methodological transparency. The inclusion of a large number of studies, a substantial total sample size, and broad geographic coverage provides valuable reference material for clinical practitioners and supports future research endeavors. Nevertheless, several limitations should be acknowledged. First, owing to the predefined inclusion and exclusion criteria, only cross-sectional studies were included, which precludes the establishment of causal relationships. Second, substantial heterogeneity was observed across the included studies. Although subgroup analyses were conducted based on characteristics such as gender, age, diagnostic criteria, geographic region, and year of study, the heterogeneity did not decrease appreciably. Consequently, the pooled prevalence estimates should be interpreted as summary indicators based on available data, rather than precise national prevalence figures for functional constipation among Chinese children and adolescents. Additionally, the number of studies examining specific influencing factors—such as gender, obesity, and household income—was limited, rendering the pooled effect sizes potentially unstable or biased; thus, these findings should be regarded as preliminary and exploratory. Furthermore, the diagnostic criteria for functional constipation employed across the included studies were not equivalent, which may have introduced bias, warranting cautious interpretation. Finally, the relatively small number of included studies and the predominance of data derived from domestic Chinese databases may limit the generalizability of the results. Future research should incorporate more high-quality studies to further validate the robustness of these findings.

## 5 Conclusion

In summary, the prevalence of functional constipation among children and adolescents in China is relatively high. Major influencing factors identified for functional constipation in this population include sex, a family history of constipation, low water intake, low intake of vegetables and fruits, Iow physical activity, being scolded for poor bowel habits in early childhood, non-breastfeeding, picky eating, food allergy, obesity, high household income, inadequate sleep, and failure to establish proper bowel habits. Therefore, it is essential to prioritize the training of primary care healthcare professionals, enabling them to effectively disseminate disease-related knowledge and education. Strengthening parental awareness of the risks associated with functional constipation in children and adolescents, implementing early prevention measures, reducing the disease burden on affected children and their families, and ultimately improving their quality of life are crucial steps forward.

## Data Availability

The original contributions presented in the study are included in the article/Supplementary material, further inquiries can be directed to the corresponding author.
