# Supplementary material for: Prevalence and influencing factors of functional constipation in Chinese children and adolescents: a systematic review and meta-analysis
Source: Front Public Health. 2026 Mar 12;14:1776863. doi: 10.3389/fpubh.2026.1776863 (PMC13017950; doi:10.3389/fpubh.2026.1776863)
Supplement: Supplementary file 1 [file Data_Sheet_1.DOCX]

# Supplementary File 1. The detailed information of literature retrieval strategy

The following is the database search strategy of this study.

**Search strategy**

P（population）：Chinese children and adolescents

I（intervention）：no interventions

C（comparison）：no comparisons

O（outcome）：Prevalence and influencing factors of functional constipation in Chinese children and adolescents

S（study）：Cross-sectional study or Retrospective study

**Search formula**

① The search on **PubMed, Web of Science, Embase, and Cochrane Library** was performed with the following search formula:

#1 Child [Mesh]

#2 Children [Title/Abstract] OR Kids [Title/Abstract] OR Kid [Title/Abstract] OR Adolescent [Mesh] OR Adolescents [Title/Abstract] OR Adolescence [Title/Abstract] OR Teens [Title/Abstract] OR Teen [Title/Abstract] OR Teenagers [Title/Abstract] OR Teenager [Title/Abstract] OR Youth [Title/Abstract] OR Youths [Title/Abstract] OR Female Adolescent [Title/Abstract] OR Female Adolescents [Title/Abstract] OR Male Adolescent [Title/Abstract] OR Male Adolescents [Title/Abstract]

#3 #1 OR #2

#4 Constipation [Mesh]

#5 Functional Constipation [Title/Abstract] OR Habitual Constipation [Title/Abstract] OR Chronic Constipation [Title/Abstract] OR Colonic Inertia [Title/Abstract] OR Colonic Inertia Dyschezia [Title/Abstract] OR Dyschezia [Title/Abstract] OR Defecation Disorders [Title/Abstract] OR Gastrointestinal Diseases [Title/Abstract] OR Gastrointestinal Disease [Title/Abstract] OR Gastrointestinal Disorders [Title/Abstract] OR Gastrointestinal Disorder [Title/Abstract] OR Functional Gastrointestinal Disorders [Title/Abstract] OR Functional Gastrointestinal Disorder [Title/Abstract] OR Cholera Infantum [Title/Abstract]

#6 #4 OR #5

#7 China [Mesh]

#8 Chinese [Title/Abstract] OR People´s Republic Of China [Title/Abstract] OR Mainland China [Title/Abstract] OR Sinkiang [Title/Abstract] OR Inner Mongolia [Title/Abstract] OR Manchuria [Title/Abstract] OR HongKong [Title/Abstract] OR TaiWan [Title/Abstract] OR MaCao [Title/Abstract]

#9 #7 OR #8

#10 Prevalence [Mesh]

#11 Prevalences [Title/Abstract] OR Point Prevalence [Title/Abstract] OR Point Prevalences [Title/Abstract] OR Period Prevalence [Title/Abstract] OR Period Prevalences [Title/Abstract] OR Epidemiology [Mesh] OR Social Epidemiology [Title/Abstract] OR Social Epidemiologies [Title/Abstract] OR Epidemics [Title/Abstract] OR Incidence [Title/Abstract] OR Morbidity [Title/Abstract] OR Outbreaks [Title/Abstract] OR Surveillance [Title/Abstract] OR Endemics [Title/Abstract] OR Occurrence [Title/Abstract] OR Frequency [Title/Abstract] OR Cross-Sectional Studies [Mesh] OR Cross Sectional Studies [Title/Abstract] OR Cross-Sectional Study [Title/Abstract] OR Cross-Sectional Survey [Title/Abstract] OR Cross-Sectional Surveys [Title/Abstract] OR Disease Frequency Survey [Title/Abstract] OR Disease Frequency Surveys [Title/Abstract] OR Cross-Sectional Analyses [Title/Abstract] OR Cross-Sectional Analysis [Title/Abstract] OR Prevalence Studies [Title/Abstract] OR Prevalence Study [Title/Abstract]

#12 #10 OR #11

#13 #3 AND #6 AND #9 AND #12

② The search on **CNKI, Wan Fang Data, SinoMed, and VIP** was performed with the following search formula:

（（儿童[主题词]）或（小儿[主题词]）或（幼儿[主题词]）或（未成年人[主题词]）或（青少年[主题词]）或（青年[主题词]））和（（便秘[主题词]）或（功能性便秘[主题词]）或（慢性便秘[主题词]）或（习惯性便秘[主题词]）或（特发性便秘[主题词]）或（功能性胃肠道疾病[主题词]）或（排便障碍[主题词]）或（排便困难[主题词]））和（（患病率[主题词]）或（检出率[主题词]）或（流行病学[主题词]）或（发生率[主题词]）或（现状[主题词]）或（调查[主题词]）或（现况[主题词]））
