# Supplementary material for: Prevalence and influencing factors of functional constipation in Chinese children and adolescents: a systematic review and meta-analysis
Source: Front Public Health. 2026 Mar 12;14:1776863. doi: 10.3389/fpubh.2026.1776863 (PMC13017950; doi:10.3389/fpubh.2026.1776863)
Supplement: Supplementary file 2 [file Table_1.DOCX]

# Supplementary File 2. The detailed results of literature evaluation

Table 1. Results of quality evaluation included in the literature

| **Study** | **Q1** | **Q2** | **Q3** | **Q4** | **Q5** | **Q6** | **Q7** | **Q8** | **Q9** | **Q10** | **Q11** | **Total score** | **Rate** |
| --- | --- | --- | --- | --- | --- | --- | --- | --- | --- | --- | --- | --- | --- |
| Zhang et al, 2010 | 1 | 1 | 1 | 1 | 0 | 1 | 1 | 1 | 1 | 1 | 0 | 9 | High |
| Zhou et al, 2010 | 1 | 1 | 0 | 1 | 0 | 1 | 1 | 1 | 0 | 1 | 0 | 7 | Middle |
| Chan et al, 2010 | 1 | 1 | 1 | 1 | 0 | 1 | 1 | 1 | 1 | 1 | 0 | 9 | High |
| Zhou et al, 2011 | 1 | 0 | 1 | 1 | 0 | 1 | 0 | 1 | 1 | 1 | 0 | 7 | Middle |
| Xiong et al, 2011 | 1 | 1 | 1 | 1 | 0 | 1 | 0 | 1 | 0 | 1 | 0 | 7 | Middle |
| Lü et al, 2012 | 1 | 1 | 0 | 1 | 0 | 1 | 1 | 1 | 0 | 1 | 0 | 6 | Middle |
| Yan et al, 2015 | 1 | 1 | 0 | 1 | 0 | 1 | 0 | 0 | 0 | 1 | 0 | 5 | Middle |
| Zhang et al, 2015 | 1 | 1 | 0 | 1 | 0 | 0 | 0 | 0 | 0 | 1 | 0 | 4 | Middle |
| Wu et al, 2016 | 1 | 1 | 0 | 1 | 0 | 1 | 0 | 1 | 1 | 1 | 0 | 7 | Middle |
| Zhao et al, 2017 | 1 | 1 | 0 | 1 | 0 | 0 | 0 | 0 | 0 | 1 | 0 | 4 | Middle |
| Ji et al, 2018 | 1 | 1 | 1 | 1 | 0 | 1 | 1 | 1 | 0 | 1 | 0 | 8 | High |
| Zhou et al, 2018 | 1 | 1 | 1 | 1 | 0 | 1 | 0 | 1 | 1 | 1 | 0 | 8 | High |
| Chen et al, 2019 | 1 | 1 | 1 | 1 | 0 | 1 | 0 | 1 | 1 | 1 | 0 | 8 | High |
| Yang et al, 2020 | 1 | 1 | 1 | 1 | 0 | 0 | 0 | 0 | 0 | 1 | 0 | 5 | Middle |
| Wang et al, 2020 | 1 | 1 | 1 | 1 | 0 | 1 | 0 | 0 | 0 | 1 | 0 | 6 | Middle |
| Huang et al, 2021 | 1 | 1 | 1 | 1 | 0 | 1 | 1 | 1 | 0 | 1 | 0 | 8 | High |
| Li et al, 2022 | 1 | 1 | 1 | 1 | 0 | 1 | 0 | 1 | 1 | 1 | 0 | 8 | High |
| Zhuang et al, 2022 | 1 | 1 | 1 | 1 | 0 | 1 | 1 | 1 | 0 | 1 | 0 | 8 | High |
| Zhang et al, 2022 | 1 | 1 | 1 | 1 | 0 | 1 | 1 | 1 | 1 | 1 | 0 | 9 | High |
| Lin et al, 2022 | 1 | 1 | 1 | 1 | 0 | 1 | 0 | 1 | 0 | 1 | 1 | 8 | High |
| Huang et al, 2022 | 1 | 1 | 1 | 1 | 0 | 1 | 1 | 1 | 1 | 1 | 0 | 9 | High |
| Yang et al, 2023 | 1 | 1 | 1 | 1 | 0 | 1 | 0 | 1 | 1 | 1 | 0 | 8 | High |
| Xu et al, 2023 | 1 | 1 | 0 | 1 | 0 | 1 | 0 | 0 | 0 | 1 | 0 | 5 | Middle |
| Zhou et al, 2023 | 1 | 1 | 1 | 1 | 0 | 1 | 1 | 1 | 1 | 1 | 0 | 9 | High |
| Wang et al, 2024 | 1 | 1 | 1 | 1 | 0 | 1 | 1 | 1 | 0 | 1 | 0 | 8 | High |
| Gong et al, 2025 | 1 | 1 | 1 | 1 | 0 | 1 | 1 | 0 | 1 | 1 | 0 | 8 | High |
| Yin et al, 2025 | 1 | 1 | 1 | 1 | 0 | 1 | 0 | 1 | 1 | 1 | 0 | 8 | High |
