# Supplementary material for: Prevalence and influencing factors of functional constipation in Chinese children and adolescents: a systematic review and meta-analysis
Source: Front Public Health. 2026 Mar 12;14:1776863. doi: 10.3389/fpubh.2026.1776863 (PMC13017950; doi:10.3389/fpubh.2026.1776863)
Supplement: Supplementary file 3 [file Table_2.DOCX]

# Supplementary File 3. Sensitivity Analysis Results for Influencing Factors

Table 2. Sensitivity analysis of influencing factors of functional constipation in Chinese children and adolescents

| **Influencing Factors** | **Fixed effect model**  **[ *OR*（95%*CI*）]** | **Random effect model**  **[ *OR*（95%*CI*）]** |
| --- | --- | --- |
| Female | 1.40（1.04，1.90） | 1.40（1.04，1.90） |
| Family history of constipation | 2.96（2.57，3.40） | 2.72（2.03，3.64） |
| Inadequate water intake | 3.36（2.74，4.11） | 3.32（2.02，5.47） |
| Low intake of vegetables and fruits | 5.32（4.68，6.05） | 4.52（1.77，11.56） |
| Iow physical activity | 1.47（1.32，4.33） | 2.75（1.19，6.35） |
| Adequate sleep | 0.58（0.38，0.88） | 0.58（0.38，0.88） |
| Being scolded for poor bowel habits in early childhood | 3.23（2.21，4.71） | 3.23（2.21，4.71） |
| Non-breastfeeding | 3.71（2.87，4.80） | 5.09（1.82，14.26） |
| Picky eating | 2.88（1.49，3.71） | 2.79（1.71，4.56） |
| Food allergy | 3.87（1.48，9.73） | 3.45（1.20，9.92） |
| Obesity | 2.28（1.33，3.89） | 2.28（1.33，3.89） |
| High household income | 1.50（1.07，2.10） | 1.50（1.07，2.10） |
| Frequent consumption of fried foods | 1.91（1.62，2.24） | 3.70（0.75，18.27） |
| Development of good bowel habits | 0.54（0.43，0.68） | 0.54（0.43，0.68） |
| Probiotic supplementation after birth | 1.86（1.03，5.63） | 2.20（0.86，5.62） |
